# Meditation affects word recognition of meditation novices

**DOI:** 10.1007/s00426-021-01522-5

**Published:** 2021-05-09

**Authors:** Larissa Lusnig, Ralph Radach, Markus J. Hofmann

**Affiliations:** Department of Psychology, University of Wuppertal, Wuppertal, Germany

## Abstract

This work represents one of the first attempts to examine the effects of meditation on the processing of written single words. In the present longitudinal study, participants conducted a lexical decision task and rated the affective valence of nouns before and after a 7-week class in mindfulness meditation, loving-kindness meditation, or a control intervention. Both meditation groups rated the emotional valence of nouns more neutral after the interventions, suggesting a general down-regulation of emotions. In the loving-kindness group, positive words were rated more positively after the intervention, suggesting a specific intensification of positive feelings. After both meditation interventions, response times in the lexical decision task accelerated significantly, with the largest facilitation occurring in the loving-kindness group. We assume that meditation might have led to increased attention, better visual discrimination, a broadened attentional focus, and reduced mind-wandering, which in turn enabled accelerated word recognition. These results extend findings from a previous study with expert Zen meditators, in which we found that one session of advanced meditation can affect word recognition in a very similar way.

## Introduction

Research on meditation has sparked substantial scientific interest in recent years. The effects of meditation practice on increased attention have generated particular interest in the research community (Jha et al., 2007; Pagnoni & Cekic, 2007; Slagter et al., 2007). Meditation practice is associated with improved efficiency in attentional processing (van den Hurk et al., 2010) and increased sustained attention (Chambers et al., 2008; MacLean et al., 2010). Experienced meditators show reduced interference in the Stroop task compared to control subjects (Chan & Woollacott, 2007) and can decelerate binocular rivalry switching (Carter et al., 2005). Further, meditation practice can lead to a reduced thought distraction and a strengthened present focus (Kok & Singer, 2017). Tang et al. (2007) found that already after a 5-day meditation training, participants achieved more improved performance on the attention network test compared to a control group.

Substantial scientific research has focused on the effects of meditation on emotion regulation. Meditation practice can reduce negative affect (Sears & Kraus, 2009), regulate symptoms of anxiety (Hofmann et al., 2010; Tang et al., 2007), stress (Goyal et al., 2014), and depression (Grecucci et al., 2015; Tang et al., 2007). At work, meditation can lead to reduced emotional exhaustion and increased job satisfaction (Hülsheger et al., 2013). In meditators, brain gray matter density is increased in the left hippocampus (Hölzel et al., 2011), which is associated with emotion regulation (Corcoran et al., 2005).

In recent years, a new field of meditation research has been explored. A study by Tarrasch et al. (2016) was one of the first to examine the effects of meditation on reading performance. They found that subjects with developmental dyslexia and ADHD demonstrated significantly fewer reading errors after a 2-month mindfulness course. Their error rate dropped by 19% compared to their original performance. In addition, they showed increased sustained attention. Another study found that intensive meditation practice can reduce mindless reading (Zanesco et al., 2016). Already after a 5-day workshop in mindfulness-based stress reduction, participants of a pilot study demonstrated a significant increase in reading speed (Rice et al., 2020). This promising field of research is still widely unexplored. Since texts often contain words with an affective content it is also important to examine the effects of meditation practice on emotional word processing. In particular, it is not clear if different meditation styles have dissociable effects on reading speed and how fast single words with different emotional valences are processed. Basic scientific research on this topic is needed. In the current literature, we find evidence that meditation can influence the processing of emotional images. Meditators demonstrate a reduced emotional reactivity to affective images. In a study by Desbordes et al. (2012) participants showed reduced amygdala activation while watching emotional images. A decreased amygdala activation suggests that the affective images had a decreased emotional impact (Davis & Whalen, 2001). Subjects with extensive meditation experience showed less interference from affective images (Ortner et al., 2007). Chau et al. (2018) found that older subjects rate affective pictures more neutral subsequent to a meditation intervention. While these studies show that meditation can affect emotional picture evaluation, the impact of meditation on affective word processing has not yet been well explored.

### Interindividual differences and word recognition

The emotional connotation of experimental word stimuli is usually assessed by valence ratings (Citron, 2012; Jacobs et al., 2015; Kissler et al., 2007; Vo et al., 2009). Based on such ratings researchers can select word material, for example, for lexical decision tasks (LDT). During the LDT, participants are presented with letter strings, which either form a word or a meaningless stimulus. Subjects must choose under pressure of time if a word is displayed or not (Meyer & Schvaneveldt, 1971; Rubenstein et al., 1970). Word stimulus properties like arousal, frequency, imageability, valence, and many others have been shown to have an impact on word recognition (Brysbaert et al., 2011; Hofmann et al., 2007; Kuchinke et al., 2007; New et al., 2006).

Interindividual differences can influence single word recognition as well. Self-secure subjects demonstrated faster identification of words expressing positive interpersonal outcomes compared to participants that are more insecure. Insecure subjects recognized words faster that expressed negative interpersonal outcomes (Baldwin et al., 1993). In a valence identification task, dysphoric subjects needed significantly more time to recognize the valence of positive words than they needed to identify the valence expressed by negative words when contrasted with non-dysphoric participants. In an LDT, non-dysphoric participants demonstrated faster recognition of negative in comparison to neutral words. Dysphoric subjects, on the other hand, responded slower to negative than to neutral words (Siegle et al., 2002). Mueller and Kuchinke (2016) linked goal-directed behavior to slower processing of fear words. Further, they tied the spontaneous eye-blink rate, which indexes dopaminergic levels, to a processing advantage for happy words. If and to what extent the processing of words may also be influenced by meditation, is so far not well understood.

### Our previous study

In a prior study, we examined if meditation has an impact on the responses to affective and neutral words in an LDT and on the valence ratings of these words (Lusnig et al., 2020). Experienced Zen meditators rated the valence of low-arousal positive and low- and high-arousal negative words more neutral, subsequent to a 90-min meditation session. In an age- and gender-matched comparison group, valence ratings did not change significantly after the comparison intervention. In the LDT, the Zen group showed an accelerated word recognition subsequent to the meditation intervention. The comparison group did not show a significant change in response times (RTs) after the comparison intervention. Because the effect of learning to meditate could not be investigated in these expert meditators, a longitudinal study, examining participants before and after they learned to meditate, is needed.

### The present study

In the present work, we adapted the design of our previous study (Lusnig et al., 2020) for a longitudinal study. Two experimental groups and a control group (CG) participated in a meditation or control class. Before and after these interventions, all groups performed an LDT and a valence-rating task, along with a brief mood-states assessment. At baseline, subjects completed assessments on concentration capacity, intelligence, and personality traits. One experimental group participated in a 7-week class in mindfulness meditation. Mindfulness meditation is a widely used meditation style, which is similar to Zen meditation. During mindfulness meditation, the practitioner develops an effortless and non-judgmental awareness of all present-moment experiences (Kabat-Zinn, 1990). The second experimental group practiced loving-kindness meditation (LKM) for 7 weeks. LKM contains the practice of empathy, first the feeling of loving kindness is directed towards oneself and then towards others (Lutz et al., 2007). The control group participated for 7 weeks in a study group. As in the previous study, we used the low-arousal positive, low- and high-arousal negative and neutral word stimuli of Hofmann et al. (2009) and Lusnig et al. (2020). The quantity of these word stimuli was doubled for the current study.

In our prior work (Lusnig et al., 2020), the Zen group responded in an LDT globally faster to the same stimulus words after the meditation intervention than before the meditation. For the LDT of the present study, we expected both meditation groups to process word stimuli faster after the meditation classes than at baseline. Particularly mindfulness meditation has been associated with increased attention (Valentine & Sweet, 1999); therefore, we anticipated the greatest change in RTs in the mindfulness group (MG). We predicted that RTs in the control group would not differ significantly when compared before and after the control intervention. In the initial study by Hofmann et al. (2009), subjects responded slower to low-arousal negative words than to neutral words. In our previous meditation work, we replicated this result in the Zen- and the control group. Consequently, for the present study, the expectation was that all three groups would process low-arousal negative words slower than neutral words.

In Lusnig et al.’s (2020) valence-rating task, the Zen group rated positive, low- and high-arousal negative words more neutral after a single 90-min meditation session. The comparison group did not demonstrate significantly different valence ratings before and after the comparison intervention. Based on these results, we expected that both meditation groups would rate emotion words more neutral after the meditation classes. Especially LKM is associated with an increased experience of positive emotions (Zeng et al., 2015); therefore, we anticipated more positive valence ratings in the loving-kindness group (LKG) than in the MG. We also predicted that the control group would not rate the valence of emotion words significantly different after the control intervention. Participants performed assessments on personality traits, concentration capacity, and intelligence to control for interindividual differences between the three groups. We did expect, however, that mood states would change after the meditation interventions in the LKG and the MG, but not in the CG.

## Methods and materials

### Participants

Thirty-nine German native speakers participated in the present study. Thirteen of them took part in the LKG (12 female, 19–39 years of age, mean age = 22.2), thirteen in the MG (11 female, 19–44 years of age, mean age = 24.5), and thirteen in the CG (12 female, 19–42 years of age, mean age = 21.8). The formula of Westfall et al. (2014) was used to calculate an appropriate sample size. Variance partitioning coefficients were estimated based on the values of our previous study (Lusnig et al., 2020). To obtain a medium effect size of *d* = 0.5 and a power of 0.8 while using 400 word-stimuli per participant, a sample size of 11.8 participants per group is required. As mentioned above, we worked with a sample size of 13 participants per group. Thirty-eight participants were right-handed one was left-handed. All subjects were students of the University of Wuppertal; they were recruited via online advertisements. Subjects were randomly assigned to the three groups. None of the participants reported prior experience with meditation or yoga. None of them had a history of psychiatric disorders or any reading and writing difficulties. All participants received course credits for participation.

### Covariates

Participants completed, at first, the “Aktuelle Stimmungsskala” (ASTS) to examine possible changes in mood states provoked by the interventions in every group. The ASTS measures subjects’ current mood states; the scales are “positive mood”, “sorrow”, “desperateness”, “fatigue” and “anger”. The internal consistency has Cronbach’s *α* values between *α* = 0.83 and 0.94. Factor analysis provided one, two and four factor-based approaches. The author provides evidence for convergent and differential validity (Dalbert, 1992a, 1992b). Participants conducted this test at baseline and after the interventions. Possible significant group differences in personality traits, sustained attention, and intelligence were examined by covariate tests, which subjects conducted at baseline. Subjects performed the d2-Revision test (d2-R), an assessment of sustained attention and the ability to focus on task. In the d2-R, subjects have to find the letter “d” with two marks while not getting distracted by similar-looking stimuli. Cronbach’s alpha values are for the scales “number of processed target objects” and for “concentration capacity” are between *α* = 0.89 and 0.95, for “percentage of errors” between *α* = 0.80 and 0.91, depending on the age group. Authors provide empirical evidence for criterion and construct validity (Brickenkamp et al., 2010). This was followed by the Multiple-Choice Vocabulary Intelligence Test (MWT-B). Here, participants have to point out a correct German word among four similar nonwords in a multiple-choice procedure. It contains 37 items, which are sorted by level of difficulty. For the MWT-B retest reliability is reported with a correlation of *r* = 0.95 after 6 months and *r* = 0.87 after 14 months. The author provides empirical evidence for criterion validity (Lehrl, 2005). At last, subjects performed the Big Five personality test (B5T), which measures personality traits as specified in the Big Five model of personality. The B5T had in the original version five scales “neuroticism” (Cronbach’s alpha, *α* = 0.90), “conscientiousness” (*α* = 0.77), “extraversion” (*α* = 0.87), “agreeableness” (*α* = 0.76) and “openness to experience” (*α* = 0.76). For the revised version, which was used in the present study, three basic requirements “need for power and influence” (*α* = 0.78), “need for safety and peace” (α = 0.84), and “need for achievement and performance” (*α* = 0.82) were added. The test demonstrates factorial validity (Satow, 2011).

### Stimuli

The stimulus set included 400 words (German nouns) and 400 altered words (nonwords). Half of the item set was taken from Hofmann et al. (2009), while the other half was generated using the same construction rules. All stimulus words were part of the revised Berlin Affective Word List (BAWL-R; Vo et al., 2009). The stimulus set contained four stimulus conditions: low-arousal negative, high-arousal negative, (low-arousal) positive, and neutral words. The stimulus condition “High-arousal positive words” could not be generated because the BAWL-R did not provide enough high-arousal positive nouns (cf. Hofmann et al., 2009; Lusnig et al., 2020; see Fig. 1 in Vo et al., 2009). Each of the four stimulus conditions of the whole item set contained 100 words. Low-arousal negative, positive and neutral words were matched for arousal. High-arousal negative words were matched for valence to low-arousal negative words, but their arousal values were maximized. Nouns were matched for the following psycholinguistic variables, which can influence LDTs: arousal, word frequency, emotional valence, number of orthographic neighbors, number of letters, imageability, mean bigram frequency (type), and mean bigram frequency (token) (see Table 1, and Table 1 in Hofmann et al., 2009). The selected nouns were modified to create nonwords. For this purpose, a vowel of a word stimulus was replaced by a consonant or another vowel. Stimuli were subdivided in Subset A and Subset B, each consisting of 200 words. To test whether the materials were matched for these variables, we conducted 2 × 4 ANOVAs (subset × emotion condition; all *F*s < 1).

**Table 1:** Mean values and standard errors of the manipulation and control variables for high-arousal negative, low-arousal negative, positive, and neutral words

Control and manipulation variables	High-arousal negative words		Low-arousal negative words		Neutral words		Positive words	
	*M*	SE	*M*	SE	*M*	SE	*M*	SE
Emotional valence	− 1.31	0.05	− 1.30	0.04	0.06	0.04	1.17	0.07
Arousal	3.90	0.03	2.83	0.04	2.82	0.03	2.82	0.03
Imageability	4.50	0.17	4.11	0.20	4.24	0.16	4.26	0.16
Number of letters	6.42	0.17	6.06	0.21	6.16	0.18	6.18	0.19
Word frequency	12.94	0.31	13.20	0.38	12.62	0.29	12.56	0.27
Number of orthographic neighbors	0.98	0.24	1.50	0.30	1.20	0.24	1.18	0.27
Mean bigram frequency (type)	3113.55	284.41	3408.68	245.04	3525.47	272.50	3223.81	277.65
Mean bigram frequency (token)	167,061.37	19,957.41	189,351.71	21,027.04	190,835.14	19,536.51	178,481.74	20,794.76

### Procedure

#### Pre-test

Subjects first completed the ASTS, then the d2-R, the MWT-B, and the B5T. Subsequently, subjects took part in the main experiment. Stimuli were presented on a 21-inch TFT display running at 70 Hz, with the distance between eyes and monitor held constant at approximately 65 cm.

Participants with even participant numbers were presented with Subset A of the stimuli set. They were asked to press key “2”, using the left index finger when identifying a nonword, and to press key “8”, using the right index finger when they recognized the stimulus as a word. After participants had processed the first half of Subset A, they were presented with the second half of subset A. To balance for RT differences due to hand dominance, subjects now pressed key “2”, using the left index finger when identifying a word, and pressed key “8”, using the right index finger when they identified a nonword. This way we also excluded the risk that possible effects were caused by the composition of one of the subsets, for instance by mood induction due to many words of the same emotion category (Niedenthal & Setterlund, 1994). Participants with uneven participant numbers were presented first with the first half of Stimulus Subset B, pressing the left index finger for a word. In the second half of Subset B, hand assignment was reversed. In any case, responses were to be executed as quickly and accurately as possible.

Before the LDT, participants were made familiar with the test by responding to five practice stimuli. The word stimuli appeared in black uppercase letters on a light gray background, in 20 pt Times New Roman font using presentation-software PsychoPy, version 1.82.01 (Peirce, 2007). Stimuli were pseudorandomized. At most three words or nonwords were presented consecutively. During each trial, for 700 ms, a fixation cross (+) was displayed, followed by a word or nonword for 1000 ms. A white screen was shown for 500 ms and then for 1500 ms a mask (#####) (see Fig. 1 in Lusnig et al., 2020). After a 5-min break, subjects rated the valence of the words, which they had seen before in the LDT. One word at a time was presented on the screen. Below every word, a seven-point grading scale from − 3 to 3 was displayed (0 = neutral, − 3 = very negative, 3 = very positive). Subjects gave their responses by clicking the respective number with the cursor. After pressing the space bar, the next word appeared on the screen. The duration of the LDT was about 20 min followed by a 5-min break, the valence rating task lasted for approximately 12 min. The whole experiment, including also covariate tests and instructions, lasted approximately 75 min.

#### Post-test

Subjects took part in the post-test about 1 week after having finished one of the meditation classes or the study group. Participants first finished the ASTS, other covariate tests were not conducted during the post-test. The instructions and procedures for the LDT were the same as the ones given in the pre-test. Participants with even numbers were presented with stimulus Subset B, subjects with uneven participant numbers completed Subset A. Participants took a 5-min break. Then, they rated the valence of the words they had seen during the post-LDT.

#### Meditation or comparison interventions

The week after the pre-test, all subjects took part in a 1.5-h intervention in the morning on the same day of the week. The training took place in 8 consecutive weeks. In the fourth week, training was suspended due to a national holiday. A local experienced meditation-trainer led both meditation groups.

#### Mindfulness meditation intervention

Participants of the MG learned at first which sitting postures are adequate for meditation, how to relax their body, and how to breathe calmly and naturally. Then, they practiced observing their thoughts, emotions, and physical feelings and tried to let them go instead of being caught up in them.

#### Loving-kindness meditation intervention

First, subjects of the LKG practiced suitable sitting postures for meditation, how to exercise a natural and calm breath, and technics to relax their body. They learned how to be aware of their thoughts, emotions, and feelings and trained to develop equanimity and self-empathy regarding these states. Gradually subjects broadened their empathy to their feelings and emotions and situations of others, also using visual imagery.

#### Control intervention

The study group aimed to involve subjects of the CG in a silent active control intervention very close to their usual activities. All participants were college students; therefore, a study group was selected as an adequate intervention. Participants were instructed to study silently for a class they were currently attending. An undergraduate assistant supervised the study group.

### Data analysis

For the LDT, RTs of the correctly given answers were analyzed using linear mixed-effects models (LMEs). 14.95% of all responses were incorrect, they were excluded from the data analysis. The models were calculated with the statistical software environment R, (version 3.4.2, http://cran.r-project.org). Specifically, we used the lme4 library with the lmer function (version 1.1–14, Bates et al., 2015). The lmer function fits an LME to the data. An LME data analysis considers participant and item variance concurrently in a non-hierarchical approach. Averaging at first level treats the error variance of items as fixed effects. Separate random intercepts for subjects and items result in treating subject and item variance more sensitively. “Subjects” and “items” were fitted as random effects. As fixed effects, we fitted “groups” (LKG/MG/CG), “time” (before/after intervention) and “emotional valence” (positive words/low-arousal negative/high-arousal negative). Fixed effects were represented with the use of effects coding. Low-arousal negative, high-arousal negative, and positive words were confronted with neutral words. We calculated a time slope for the random effect “item” because the time-series effect might be different for different items (Baayen et al., 2008). For every group, separate LMEs were calculated to examine the origin of significant interactions. The dependent variable “response time” was log-transformed to satisfy the assumption of normality of the residuals, which were verified by qqplots. Estimates of the regression coefficients, their standard errors, and t values are reported for the LMEs. P values are reported on the basis of the Satterthwaite approximation, which is implemented in the lmerTest package, (Version 2.0-36, Kuznetsova et al., 2017).

Regarding the valence rating experiment, we examined responses given on a seven-point grading scale ranging from − 3 to 3. An LME was used again to analyze the experimental data. The procedure of the data analysis was the same as for the response time experiment. Data points, which were not in a range between – 3 and 3 standard deviations of the residual error, were discarded from the calculations (approximately 1% of the data). To analyze the results of the ASTS we conducted a 3 × 2 × 5 ANOVA with the factors “group”, “time” and “mood conditions”.

## Results

### Covariates

To control for a possible change in mood induced by the interventions, subjects completed the ASTS at baseline and after interventions. Mood did not change significantly over time points in any of the groups. Mood conditions (positive mood, sorrow, desperateness, fatigue, and anger) were rated significantly different (see Table 2). This effect demonstrates, for example, the difference between positive mood and sorrow; for the discussed effects of the present study, however, it does not have relevance. In addition, subjects were tested at baseline for group differences in personality traits, intelligence, and concentration capacity to control for influences of these factors on the RT results. In these comparisons, no significant group differences were found. All statistical values for group differences, mean values, and standard deviations of the baseline covariate tests are reported in Table 3.

**Table 2:** Statistical group differences and mean squared errors (MSE) of ASTS (tested at baseline and after intervention)

	Statistical group differences
Time	*F*(1, 36) = 2.31, *p* = 0.14, MSE = 4.13
Time: Group	*F*(2, 36) = 2.39, *p* = 0.11
Mood conditions	*F*(1, 36) = 496.85, *p* = 0.00, MSE = 54.32
Mood conditions: Group	*F*(2, 36) = 1.04, *p* = 0.39
Time: Mood conditions	*F*(2, 36) = 0.29, *p* = 0.78, MSE = 26.47
Time: Mood conditions: Group	*F*(2, 36) = 0.61, *p* = 0.66

Time (before/after intervention), Group (LKG/MG/CG), mood conditions (positive mood, sorrow, desperateness, fatigue, and anger)

**Table 3:** Baseline covariate tests: statistical group differences, mean squared errors (MSE), mean values, and standard deviations

Covariate test	Statistical group differences	LKG		MG		CG	
		*M*	SD	*M*	SD	*M*	SD
**Big Five personality test**							
Neuroticism	*F*(2, 36) = 2.37, *p* = 0.11, MSE = 2.01	4.85	1.86	4.77	1.34	3.77	1.01
Extraversion	*F*(2, 36) = 0.46, *p* = 0.63, MSE = 3.16	5.69	1.60	5.77	2.39	6.31	1.11
Conscientiousness	*F*(2, 36) = 1.30, *p* = 0.29, MSE = 4.39	4.23	1.92	5.54	2.15	4.69	2.21
Agreeableness	*F*(2, 36) = 1.18, *p* = 0.32, MSE = 2.41	5.15	1.68	5.92	1.71	6.00	1.23
Need for safety and peace	*F*(2, 36) = 2.96, *p* = 0.06, MSE = 1.74	4.62	1.19	5.46	1.56	4.23	1.16
Need for power and influence	*F*(2, 36) = 0.71, *p* = 0.50, MSE = 2.86	4.54	1.81	4.31	1.55	5.08	1.71
Openness	*F*(2, 36) = 2.83, *p* = 0.07, MSE = 2.07	5.77	1.59	5.38	1.04	4.46	1.61
Need for achievement and performance	*F*(2, 36) = 0.01, *p* = 0.99, MSE = 3.03	5.00	1.87	5.08	1.89	5.00	1.41
**d2-Revision, test concentration capacity**							
Number of processed target objects	*F*(2, 36) = 2.18, *p* = 0.13, MSE = 465.78	163.77	22.06	179.00	21.72	179.15	20.96
Concentration capacity	*F*(2, 36) = 1.91, *p* = 0.16, MSE = 666.45	143.38	28.31	161.38	28.47	159.53	19.68
Percentage of errors	*F*(2, 36) = 1.89, *p* = 0.17, MSE = 22.52	11.37	4.72	8.24	4.35	11.37	5.14
**Intelligence test (MWT-B)**	*F*(2, 36) = 2.05, *p* = 0.14, MSE = 6.78	22.23	2.52	23.37	2.36	21.31	2.89

### Lexical decision and valence rating data

**Fig. 1:**
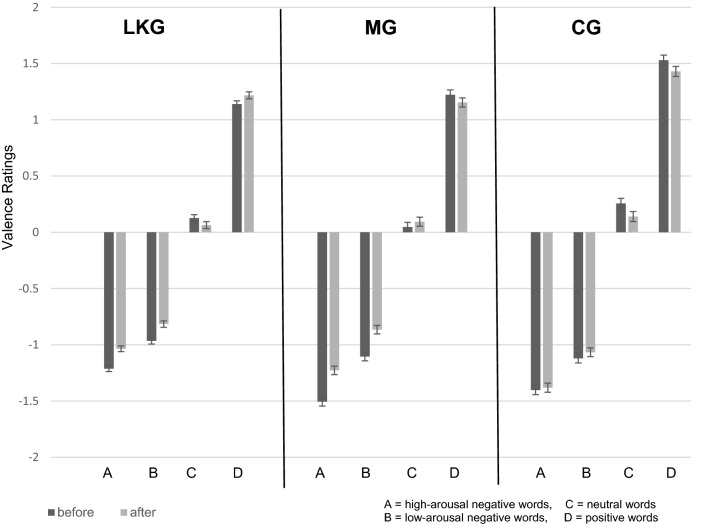
Results of the valence rating experiment. Error bars indicate standard errors

**Fig. 2:**
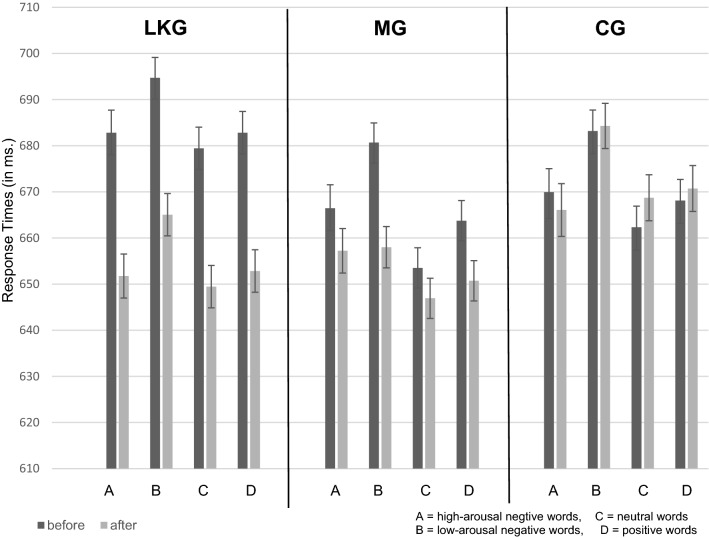
Results of the lexical decision experiment. Error bars indicate standard errors

Figure 1 shows that after the loving-kindness- and the mindfulness-meditation sessions affective valence ratings were more neutral, except for valence ratings to positive words in the LKG, which became more positive. Figure 2 shows that RTs in the LDT were shorter after both meditation interventions, but not after the control intervention. The LME analysis revealed for the valence rating as well as for lexical decision data significant interactions of “group” and “time” (see Table 4 for entire analysis). The valence rating data showed for “time” as well as for “group” significant two-way interactions with “positive”, “high-arousal negative”, and “low-arousal negative” words. Further, we found significant main effects for “high-arousal negative”, “low-arousal negative” and “positive” words. The analysis of the lexical decision data revealed for "low-arousal negative" words a significant main effect.

**Table 4:** Valence rating and LDT Experiments: estimates of regression coefficients, their standard errors, *t* values, *p* values, and Cohen's *d* effect sizes of the overall analyses

	Valence ratings					Lexical decision task				
	*B*	SE	*t*	*p*	*d*	*B*	SE	*t*	*p*	*d*
Time	0.013	0.024	0.54	0.592	0.01	– 0.002	0.004	– 0.55	0.586	– 0.02
Group	– 0.009	0.032	– 0.28	0.783	– 0.15	0.006	0.016	0.41	0.688	0.13
High-arousal negative	– 1.273	0.052	– 24.57	0.001***	– 1.91	0.001	0.008	0.01	0.997	0.01
Low-arousal negative	– 0.933	0.052	– 18.01	0.001***	– 1.40	0.026	0.008	3.28	0.001**	0.27
Positive	1.739	0.052	33.59	0.001***	2.64	– 0.006	0.008	– 0.82	0.412	– 0.07
Time:group	0.039	0.015	2.70	0.007**	0.04	– 0.022	0.003	– 8.21	0.001***	– 0.15
Time:high-arousal negative	0.084	0.042	2.00	0.045*	0.08	– 0.006	0.006	– 0.96	0.335	– 0.04
Group:high-arousal negative	0.128	0.018	7.08	0.001***	0.09	– 0.001	0.003	– 0.35	0.730	– 0.01
Time:low-arousal negative	0.106	0.042	2.53	0.011*	0.09	– 0.001	0.006	– 0.14	0.890	– 0.01
Group:low-arousal negative	0.102	0.018	5.66	0.001***	0.07	– 0.003	0.003	– 0.85	0.394	– 0.02
Time:positive	– 0.106	0.042	– 2.53	0.011*	– 0.11	– 0.002	0.006	– 0.31	0.758	– 0.01
Group:positive	– 0.180	0.018	– 9.95	0.001***	– 0.13	0.001	0.003	0.30	0.768	0.01
Time:Group:high-arousal negative	0.017	0.026	0.68	0.499	0.01	0.004	0.005	0.89	0.373	0.02
Time:Group:low-arousal negative	– 0.021	0.026	– 0.82	0.412	– 0.01	0.001	0.005	0.08	0.933	0.01
Time:Group:positive	0.037	0.026	1.44	0.149	0.02	– 0.001	0.005	– 0.28	0.777	– 0.01

“***”*p* < 0.001, “**”*p* < 0.01, “*”*p* < 0.05

Separate LMEs were subsequently calculated for all three groups to resolve the significant interactions of “time” and “group” (cf. Table 5). For the valence rating and lexical decision data, we found significant main effects for “time” in the LKG and the MG, but not in the CG.

**Table 5:** Valence rating and LDT experiments: estimates of regression coefficients, their standard errors, t values, p values, and Cohen’s d effect sizes for the LKG, MG, and CG

	Valence ratings					Lexical decision task				
	*B*	SE	*t*	*p*	*d*	*B*	SE	*t*	*p*	*d*
**LKG**										
Time	0.078	0.026	3.08	0.002**	0.31	– 0.048	0.004	– 12.41	0.001***	– 0.41
High-arousal negative	– 0.980	0.051	– 19.22	0.001***	– 1.92	– 0.003	0.008	– 0.32	0.747	– 0.03
Low-arousal negative	– 0.728	0.051	– 14.27	0.001***	– 1.43	0.016	0.008	1.98	0.048*	0.20
Positive	1.361	0.051	26.69	0.001***	2.67	– 0.004	0.008	– 0.55	0.585	– 0.06
Time:high-arousal negative	0.094	0.044	2.12	0.035*	0.21	– 0.001	0.007	– 0.21	0.833	– 0.01
Time:low-arousal negative	0.053	0.044	1.20	0.231	0.12	0.006	0.007	0.81	0.417	0.03
Time:positive	– 0.011	0.044	– 0.25	0.801	– 0.03	– 0.004	0.007	– 0.57	0.569	– 0.02
**MG**										
Time	0.125	0.025	4.91	0.001***	0.19	– 0.022	0.004	– 5.96	0.001***	– 0.62
High-arousal negative	– 1.172	0.054	– 21.78	0.001***	– 2.14	0.001	0.008	0.01	0.993	0.01
Low-arousal negative	– 0.768	0.054	– 14.28	0.001***	– 1.41	0.027	0.008	3.49	0.001***	0.35
Positive	1.554	0.054	28.90	0.001***	2.85	– 0.005	0.008	– 0.70	0.484	– 0.07
Time:high-arousal negative	0.157	0.044	3.56	0.004***	0.14	– 0.005	0.006	– 0.78	0.435	0.08
Time:low-arousal negative	0.115	0.044	2.60	0.009**	0.10	– 0.013	0.006	– 1.94	0.053	– 0.19
Time:positive	– 0.194	0.044	– 4.40	0.001***	– 0.17	– 0.002	0.006	– 0.27	0.785	– 0.03
**CG**										
Time	– 0.026	0.027	– 0.95	0.342	– 0.03	– 0.001	0.004	– 0.33	0.739	– 0.03
High-arousal negative	– 1.215	0.056	– 21.65	0.001***	– 2.14	– 0.003	0.008	– 0.33	0.745	– 0.03
Low-arousal negative	– 0.930	0.056	– 16.57	0.001***	– 1.64	0.023	0.008	2.78	0.006**	0.29
Positive	1.707	0.056	30.40	0.001***	3.00	– 0.004	0.008	– 0.53	0.594	– 0.06
Time:high-arousal negative	0.047	0.047	1.01	0.311	0.03	– 0.008	0.007	– 1.23	0.221	– 0.13
Time:low-arousal negative	0.077	0.047	1.66	0.098	0.05	0.006	0.007	0.90	0.368	0.09
Time:positive	– 0.050	0.047	– 1.06	0.287	– 0.03	– 0.003	0.007	– 0.52	0.603	– 0.06

“***”*p* < 0.001, “**”*p* < 0.01, “*”*p* < 0.05

## Discussion

In the present study, we did not find any baseline differences in intelligence, concentration capacity, and personality traits between the three groups. Contrary to our expectations, mood changes could not be detected in the ASTS after any of the three interventions. Looking at word items, both meditation groups rated valence more neutral after the meditation interventions. Participants in the LKG, however, rated positive words more positively after the intervention. In the control group, valence ratings did not differ significantly after control intervention. Concerning the LDT, both meditation groups demonstrated faster word recognition after the interventions. This effect was most pronounced for participants in the LKG. The control group did not show significantly different RTs after the intervention. Hofmann et al. (2009) and our previous study found that low-arousal negative words were processed slower than neutral words. We replicated this effect in all three groups.

### The valence rating experiment

Valence ratings differed significantly after the interventions in both meditation groups. After the control intervention, valence ratings did not change. Figure 1 demonstrates that the MG and the LKG rated words more neutral after the meditation classes. The LKG, however, rated positive words more positively after the meditation intervention. These results are in line with previous work on meditation and emotion regulation. Several studies showed that particularly mindfulness meditation can down-regulate negative emotions such as anxiety (Hofmann et al., 2010; Tang et al., 2007), stress (Goyal et al., 2014), and depression (Grecucci et al., 2015; Tang et al., 2007). The practice of LKM has also been associated with increased positive emotions (Fredrickson et al., 2008; Hutcherson et al., 2008; Zeng et al., 2015). However, contrary to our expectations, we did not find evidence for changes in mood states after any of the three interventions. The reason for this could be that the mood assessment we used (ASTS) might not be sensitive or specific enough to capture the emotion regulation produced by meditation. On the other hand, these results may indicate that meditation practice does not always lead to a reduced experience of emotions. The practitioners may still experience the emotions the way they did before but do not judge them and do not get carried away by them. The fact that we did not find changes in mood states, however, ensures that the neutralized valence ratings occurred due to the meditation interventions and were not influenced by a momentary mood change.

In our previous study (Lusnig et al., 2020), adept Zen meditators assigned to words significantly more neutral valence ratings after a 90-min Zen meditation. In the previous comparison group, valence ratings did not change after the comparison intervention. These results are in line with our findings for the MG in the present study. The similarity of these results was to be expected because Zen meditation and mindfulness meditation are comparable styles of meditation. Both meditation styles belong to the category of open monitoring meditation (OMM) (Lutz et al. 2008), during which the meditator monitors, in a non-judgmental way, everything that occurs in his moment-to-moment experience, such as sounds, thoughts that pass the mind, smells, or feelings. According to Lutz et al. (2007), LKM can be seen as a special case of OMM because it contains the “cultivation of objectless awareness” and “non-referential compassion”. However, it contains also phases of focused attention meditation (FAM), during which the meditator keeps the attention all the time on one object. In the case of LKM, this object is the feeling of loving-kindness, which is directed towards oneself or other single persons (Lutz et al., 2007; Vago & Silbersweig, 2012). In the present work, the LKG rated positive words more positively after meditation. In the MG and the Zen group of the previous study, we did not find such an effect. LKM differs from mindfulness- and Zen meditation because it contains the practice of empathy and positive feelings towards others. This difference in meditation practice may have led to the more positive valence ratings in the LKG. A study by Hunsinger et al. (2013) found results similar to our study. In their work, loving-kindness novices associated significantly more positivity with neutral stimuli after a meditation intervention compared to control participants.

### The lexical decision experiment

Half of the stimulus set used in the present study was identical to the one used by Hofmann et al. (2009). They found, among other results, low-arousal negative words being processed slower than neutral words. In the present study, we replicated this effect in all three groups. In our previous work (Lusnig et al., 2020), we obtained this result in the Zen and the control group. In the current literature, it is discussed if positive or negative visual stimuli are processed more rapidly. For example, Öhman et al. (2001) found that threatening faces are processed faster than friendly faces. On the other hand, a study by Becker et al. (2012) demonstrated that dynamic happy facial expressions are detected faster than dynamic angry facial expressions. Hofmann et al. (2009) found that the arousal level affects the processing speed of emotional single words. In their study, high-arousal negative words and positive words are processed faster than low-arousal negative words. In the present study, we found the same descriptive result pattern in all three groups (see Fig. 2). The MG, however, demonstrates after the meditation intervention no difference in processing speed for high-arousal negative, low-arousal negative, and positive words (see Fig. 2). This might be because the profound practice of equanimity in mindfulness meditation minimizes the difference in arousal level for negative words.

In both meditation groups of the present study, but not the control group, RTs changed significantly after the interventions. As illustrated in Fig. 2, RTs to emotional words were faster after both meditation interventions. These results are also in accordance with those of our previous study, in which the Zen group demonstrated a significantly faster word recognition after a 90-min meditation session. Meditation is associated with increased attention (Carter et al., 2005; Chambers et al., 2008; MacLean et al., 2010; Chan & Woollacott, 2007; van den Hurk et al., 2010). Hence, it appears plausible to conclude that increased attentional resources in the meditation groups may have led to accelerated word recognition. As an alternative account, the shorter RTs could be associated with reduced mind-wandering as a result of meditation. Using functional MRI, Brefczynski-Lewis et al. (2007) found that expert meditators showed less brain activation in the default mode network. The default mode network is associated with discursive thoughts. Similarly, a study by Pagnoni et al. (2008) found in meditators decreased neural activity in default mode network regions. These authors propose that meditators may be able ‘to control the automatic cascade of semantic associations’ better than control subjects (Pagnoni & Cekic, 2007, p. 1). Therefore, spontaneous mind-wandering could be regulated more easily. In the present study, regulated mind-wandering may have helped the participants of the meditation groups to focus more closely on the current word stimulus, enabling faster responses.

Improved visual discrimination could be another process that may have contributed to faster RTs in the MG and the LKG. Expert meditators demonstrate visual attentional processing, which is more accurate and flexible in contrast to control subjects’ visual processing. For example, meditators notice changes in flickering scenes faster than controls (Hodgins & Adair, 2010). Brown et al. (1984) tested Buddhist meditators before and after a 3-month meditation retreat for visual sensitivity. After the meditation intervention, meditators noticed shorter single-light flashes and could differentiate better successive light-flashes than before the retreat. A control group did not show any such changes in visual sensitivity. In a study by MacLean et al. (2010), meditation novices improved after a 3-month meditation training visual discrimination, perceptual sensitivity, and increased vigilance during visual attention. This evidence points to the possibility that in the present study meditation training might have led to improved visual sensitivity and discrimination performance, which in turn allowed for faster responses in the LDT.

We expected that the MG would show the largest decrease in RTs after intervention because especially mindfulness meditation has been associated with increased attention (Semple, 2010; Valentine & Sweet, 1999). There was indeed a substantial response acceleration, but in our data, this effect was even more pronounced in the LKG. These results might be explained considering the association between meditation styles and narrow or broad attentional focus. Lippelt et al. (2014) argued that FAM, which contains mainly a strong concentration on a single object, leads to a narrow focus of attention. During OMM the meditator monitors all experiences non-judgmentally. This meditation style is, therefore, thought to lead to a broad attentional focus. Such a broadened attentional focus was shown to promote better performance on an attention task (Willems & Martens, 2016). In the present study, a broad attentional focus, induced by the mindfulness meditation, might have led to a more effective and therefore faster word processing. The practice of LKM contains open monitoring, the main goal of this meditation style is to broaden the feeling of loving-kindness starting from a person we like to everyone. This broadened attentional focus combined with the strong cultivation of loving-kindness might have helped the LKG to process the emotional words especially fast. Since positive affect is also associated with a broader attentional focus (Fredrickson & Branigan, 2005), the feeling of loving-kindness might have given an additional speed boost in the LKG. It would be very interesting for subsequent research to compare not just the influence of mindfulness meditation and LKM on word processing but also the effects of FAM. This way the effects of narrowed and broadened attentional focus could be compared.

### Future directions and limitations

Concerning the sequence of experimental and covariate tests in the present study, it might have been better to give to the participants first the ASTS, then the LDT and the valence rating task, and at last the remaining covariate tests. This way the mood states, which were measured with the ASTS could not have been changed through tiring covariate tests. However, since in the present study the ASTS results were not influenced by the meditation intervention, the test plays a minor role in the interpretation of our study, and we do not see problems with the sequence of the tests.

For future studies on the influence of meditation and visual word processing it might also be beneficial to use assessments on emotion regulation and increased attention not just before the meditation intervention, as in the present study, but also after it. With such an experimental design it could be extensively examined which of these underlying mechanisms of meditation influences altered word processing in meditation practitioners the most. It would be especially informative to investigate further the role of emotional variability. We would suggest that subjects should conduct adequate emotion assessments at baseline, two times during the intervention and after it.

The present study and our previous study (Lusnig et al., 2020) focused on the question if meditation practice can influence visual single word processing. Since we found that meditation practice can accelerate the responses to single words and neutralize the valence ratings of emotion words, it would be interesting to examine in the future if meditation might have similar effects on the processing of entire written texts. If the effects of meditation practice on written texts are similar to those on single words, it can be assumed that meditation can accelerate the reading speed of practitioners, as suggested also by a pilot study by Rice et al. (2020). It seems also important to examine clearly how meditation styles lead to a narrow or broad attentional focus and how such a focus affects word and text processing. Lippelt et al. (2014) proposed that FAM leads to a narrow attentional focus and that OMM induces a broadened attentional focus. It seems important to examine clearly in experimental studies how these attentional foci affect word and text processing. The further examination of narrow and broad attentional foci, triggered by meditation, could also have important implications for the treatment of anxiety disorders. Richards et al. (2014) claimed that anxious persons, who show hypervigilance, demonstrate a broadened attentional focus that scans for potential threats in the environment. The practice of FAM could be helpful for these individuals to deliberately narrow their focus on an object like the breath and calm themselves down. On the other hand, Richards et al. (2014) argued that people with anxiety disorders show, in confrontation with a specific threatening stimulus, a narrow attentional focus. OMM could help persons, in such situations, to deliberately broaden their attention and thereby allow them to detach their attention from the threatening stimulus.

## Conclusions

In a previous study Lusnig et al. (2020), we found that advanced Zen meditation can neutralize valence ratings of emotional words and accelerated RTs to these words. In the present study, we were able to obtain similar results with a longitudinal study design. Subjects, which participated in a 7-week loving-kindness- or mindfulness course, demonstrated significantly more neutral valence ratings after the interventions. Positive words were rated more positively after the LKM course. These results suggest that different meditation styles can contribute to the down- and up-regulation of emotions. However, contrary to our expectations, mood states did not appear to change after meditation interventions. In both meditation groups, RTs were faster after the interventions than before, with the largest changes occurring in the LKG. Improved increased attention, visual discrimination, and reduced mind-wandering, caused by meditation, may have enabled accelerated word recognition. The results of the present study could help to understand better the influence of meditation in text processing of affectively loaded content.

## Data Availability

The datasets analyzed during the present study are available from the corresponding author on reasonable request. The code used for the data analysis of the present study is available from the corresponding author on reasonable request.
